# Experience With the Use of an Online Community on Facebook for Brazilian Patients With Gestational Trophoblastic Disease: Netnography Study

**DOI:** 10.2196/10897

**Published:** 2018-09-24

**Authors:** Marisa Victoria Diniz, Sue Y Sun, Claudia Barsottini, Mauricio Viggiano, Roney C Signorini Filho, Bruna Sanches Ozane Pimenta, Kevin M Elias, Neil S Horowitz, Antonio Braga, Ross S Berkowitz

**Affiliations:** 1 Obstetrics Escola Paulista de Medicina Universidade Federal de São Paulo São Paulo Brazil; 2 Health Informatics Escola Paulista de Medicina Universidade Federal de São Paulo São Paulo Brazil; 3 Goiania Trophoblastic Disease Center Goiania Brazil; 4 Division of Gynecologic Oncology Obstetrics and Gynecology Harvard Medical School Boston, MA United States; 5 Brigham and Women's Hospital Boston, MA United States; 6 Rio de Janeiro Federal University Rio de Janeiro Brazil; 7 Fluminense Federal University Niteroi Brazil

**Keywords:** gestational trophoblastic disease, social media, Facebook, mobile phone

## Abstract

**Background:**

The term gestational trophoblastic disease (GTD) includes both complete and partial moles, which are uncommon nonviable pregnancies with the potential to evolve into a malignancy known as gestational trophoblastic neoplasia. While highly curable, the potential for malignancy associated with molar pregnancies worries the patients, leading them to seek information on the internet. A Facebook page headed by Brazilian specialized physicians in GTD was created in 2013 to provide online support for GTD patients.

**Objective:**

The objective of our study was to describe the netnography of Brazilian patients with GTD on Facebook (FBGTD) and to evaluate whether their experiences differed depending on whether they received care in a Brazilian gestational trophoblastic disease reference center (BRC) or elsewhere.

**Methods:**

This was a cross-sectional study using G Suite Google Platform. The members of FBGTD were invited to participate in a survey from March 6 to October 5, 2017, and a netnographic analysis of interactions among the members was performed.

**Results:**

The survey was answered by 356 Brazilian GTD patients: 176 reference center patients (RCP) treated at a BRC and 180 nonreference center patients (NRCP) treated elsewhere. On comparing the groups, we found that RCP felt safer and more confident at the time of diagnosis of GTD (*P*=.001). RCP were more likely to utilize FBGTD subsequent to a referral by health assistants (*P*<.001), whereas NRCP more commonly discovered FBGTD through Web searches (*P*<.001). NRCP had higher educational levels (*P*=.009) and were more commonly on FBGTD for ≥ 6 months (*P*=.03). NRCP were more likely to report that doctors did not adequately explain GTD at diagnosis (*P*=.007), had more doubts about GTD treatment (*P*=.01), and were less likely to use hormonal contraception (*P*<.001). Overall, 89% (317/356) patients accessed the internet preferentially from home and using mobile phones, and 98% (349/354) patients declared that they felt safe reading the recommendations posted by FBGTD physicians.

**Conclusions:**

This netnographic analysis of GTD patients on FBGTD shows that an Web-based doctor-patient relationship can supplement the care for women with GTD. This resource is particularly valuable for women being cared for outside of established reference centers.

## Introduction

The term gestational trophoblastic disease (GTD) describes a group of placental neoplasms, including both benign forms, partial and complete hydatidiform moles, and malignant forms, collectively referred to as gestational trophoblastic neoplasia (GTN). The latter includes invasive moles, choriocarcinoma, placental site trophoblastic tumors, and epithelioid trophoblastic tumors [1]. Partial and complete moles are nonviable pregnancies that progress to GTN in around 1%-5% and 20% of cases, respectively [1-5]. Patients with partial and complete moles have a particularly unfortunate situation: the happiness of a desired pregnancy is suddenly replaced with mourning a pregnancy loss complicated with the additional burden of a potentially life-threatening diagnosis [6]. While most cases of GTN follow molar pregnancies, GTN can occur after any gestational event, including ectopic and term pregnancies, sometimes delaying diagnosis and worsening prognosis [1].

In Brazil, 1 case of GTD occurs in every 200-400 pregnancies [7]. In this continent-spanning country, with 208 million inhabitants [8] distributed over a large territorial area of 8,516,000 km^2^, the treatment of women with GTD in reference centers is a challenge, especially outside state capital cities [9]. In 2013, the International Society for the Study of Trophoblastic Disease supported the development of the Brazilian Association of Gestational Trophoblastic Disease (Brazilian Association of GTD) [10]. Since then, this society has been working with health professionals all over Brazil to improve GTD treatment. Currently, there are 38 Brazilian gestational trophoblastic disease reference centers (BRC), with more than 25,000 cases of GTD treated to date and about 1500 new cases seen each year. This medical care is linked to the Brazilian public health care system and is free for patients. This has led to major advances both in the care of patients with GTD and in research on this disease in Brazil [10]. Earlier diagnosis of molar pregnancy and close postmolar follow-up using human chorionic gonadotropin (hCG) monitoring to promptly recognize GTN [11] and initiate appropriate treatment with chemotherapy in reference centers has led to dramatic decreases in maternal morbidity and mortality associated with GTD [9,12,13].

To supplement the centralized coordination of care and collaboration among BRC, the Brazilian Association of GTD created an online community on Facebook (Facebook group of the Brazilian Association of GTD; FBGTD) in 2013. This online community supports patients, patients’ relatives, and health care professionals by providing advice about GTD and helping direct patients to treatment at BRC. While there are few well-documented health care initiatives on Facebook, several Web-based communities focused on disseminating scientific data and combating false information have been shown to improve medical knowledge and the quality of patient care [14,15]. Facebook readily facilitates the exchange of information and experiences among patients, especially those with rare diseases. This exchange creates valuable relationships that support and empower patients in a way that was impossible before the social media era [16].

Careful observation and study of these Web-based relationships, called netnography, can reveal fears, feelings of unsafety, and unusual behaviors among Facebook users that may not be noted via other methods, such as a questionnaire [17]. A netnographic study has the potential to offer information about patients that may be useful to improve their care. The aim of this study was to document the netnography of Brazilian patients participating on FBGTD and evaluate how the experiences discussed or reported by patients differed depending on whether they received care within or outside of a BRC.

## Methods

### Study Design

This was a cross-sectional study using G Suite Google Platform and a netnographic analysis of interactions among patients with GTD who were members of FBGTD and were invited to participate in a survey from March 6 to October 5, 2017.

### Population

On October 5, 2017, the FBGTD group had 5783 members. Among these members, 807 were identified as GTD patients and comprised the study population. The others were excluded for the following reasons: 92 members were identified as patients’ relatives, 2990 were professionals or students from health-related areas, and the others could not be determined due to missing information. Due the nature of this research, this was a convenience sampling, not a probabilistic one.

### The Questionnaire

The questionnaire (Multimedia Appendix 1) comprised 33 questions evaluating internet use, GTD history including location of treatment (BRC or not), doctor-patient relationship, emotional well-being, and socioeconomic profile. Patients could answer the survey only after reading and agreeing to the informed consent document approved by the Ethics Review Board of Universidade Federal de São Paulo under protocol number 0019/2017. Group members easily accessed the survey from the first page of FBGTD, where it was prominently displayed.

From March 6 to April 5, 2017, there was no direct encouragement to patients to respond to the questionnaire. From April 6 to June 10, 2017, the principal investigator, MVD, who presented herself as a nurse from one of the BRCs, sent short message service (SMS) messages inviting patients who had not yet answered to complete the survey. On June 11, 2017, one of the moderators of the FBGTD group (AB) “tagged” patients who had not yet answered, inviting them to participate one last time. We made no further contact with the members of the FBGTD group. Over time, questionnaire responses progressively declined. Survey collection ended on October 5, 2017.

The principal investigator spent at least 3 hours per day observing the FBGTD over the study period to collect data about the pattern of interactions among patients as well as between patients and physicians. These interactions occurred via posts, written comments to posts, or acknowledged “likes” to posts (“thumbs up”). She qualitatively documented any recurrent use of language, symbols, or behavior common among the members of FBGTD.

### Statistical Analysis

Patients who answered the survey were grouped into 2 sets for statistical analysis: treated at a reference center (RCP) and not treated at a reference center (NRCP). Quantitative variables were analyzed using mean, SD, median, minimum and maximum, and total valid observations. Quantitative variables were compared using the nonparametric Mann–Whitney test. Qualitative variables were analyzed using frequency and percentage. To compare the groups regarding qualitative variables, we used the chi-square test, Fisher exact test, or likelihood ratio test, as indicated. The level of significance was set at *P*<.05 for all tests. The statistical analysis was conducted using IBM SPSS Statistics version 22 (IBM Corp., New York, USA).

## Results

**Figure 1:**
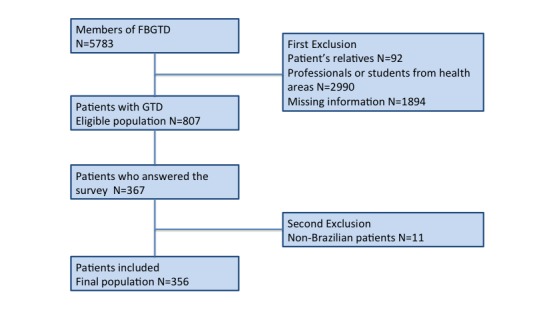
Flowchart summarizing the derivation of the study population. GTD: gestational trophoblastic disease, FBGTD: Facebook group of the Brazilian Association of GTD.

Of the 807 GTD patients who were members of the FBGTD and, therefore, eligible to participate in this study, 367 answered the questionnaire. An additional 11 non-Brazilian women were excluded, leaving 44.1% (356/807) participants who comprised the final population of Brazilian GTD patients, as shown in Figure 1. In the first phase of questionnaire responses, in which no direct encouragement to patients to respond was made, 52.2% (186/356) subjects participated in the study. After sending an SMS text message inviting participants to the study, 23% (82/356) new patients responded to the questionnaire. Finally, an additional 24.7% (88/356) only participated in the study after the request of one of the FBGTD moderators.

Demographic characteristics of patients in the FBGTD showed no differences between 176 RCP and 180 NRCP as shown in Table 1. Notably, the proportion of women living in a city with a reference center was not significantly different between patients treated (79/176) and not treated (70/180; *P*=.25) at a BRC.

When analyzing the socioeconomic characteristics of the participating members of FBGTD (Table 2), NRCP exhibited higher education levels than RCP (postgraduate education: 53/180 vs 32/176; *P*=.009). There were no differences in complaints about work problems between the groups; furthermore, there were no differences in the number of employed women between RCP (115/176) and NRCP (117/180; *P*=.94).

**Table 1:** Demographic characteristics of the study population by site of gestational trophoblastic disease care.

Characteristics		Treated at reference centers (n=176)	Not treated at reference centers (n=180)	*P* value^a^
**Age (years), n=354^b^, n (%)**				.43
	<20	11 (6.3)	6 (3.4)	
	20-39	150 (85.7)	159 (88.8)	
	≥ 40	14 (8.0)	14 (7.8)	
**Brazilian region, n (%)**				.50
	North	4 (2.3)	9 (5.0)	
	Northeast	24 (13.6)	22 (12.2)	
	Southeast	102 (58)	97 (53.9)	
	Northwest	23 (13.1)	21 (11.7)	
	South	23 (13.1)	31 (17.2)	
**Ethnic origin**, **n=352^c^, n (%)**				.77
	White	94 (54.7)	101 (56.4)	
	Black	13 (7.6)	13 (7.3)	
	Mulatto	54 (31.4)	58 (32.4)	
	Asian	11 (6.4)	7 (3.9)	
Live in a city that has a reference center for gestational trophoblastic disease, n (%)		79 (44.9)	70 (38.9)	.25

^a^Chi-square test.

^b^Two patients did not indicate age.

^c^According to the Brazilian Institute of Geography and Statistics. Ethnic-racial characteristics of the population. Classifications and identities 2013 [18]. Three patients chose not to declare ethnicity or race, and 1 Indian participant was excluded.

**Table 2:** Socioeconomic and work characteristics of women with gestational trophoblastic disease and members of Facebook group of the Brazilian Association of gestational trophoblastic disease.

Characteristics		Treated at reference centers (n=176)	Not treated at reference centers (n=180)	*P* value
**People living in the participant’s house besides her, median^a^**				.14^b^
	≤1, n (%)	67 (38.1)	54 (30.0)	.27^c^
	2-3, n (%)	87 (49.4)	100 (55.6)	—^d^
	≥4, n (%)	22 (12.5)	26 (14.4)	—
**Number of people contributing financially to the sustenance of participant’s home, median^e^**				.44^b^
	≤ 2, n (%)	160 (90.9)	159 (88.3)	.43^c^
	≥ 3, n (%)	16 (9.1)	21 (11.7)	—
Participants being paid for work during the treatment of gestational trophoblastic disease, n (%)		115 (65.3)	117 (65.0)	.95^c^
Participants reporting some work difficulties to follow their treatment (N=232), n (%)		9 (5.1)	12 (6.7)	.53^c^
Participants for whom vouchers or medical certificates have been refused, n (%)		3 (1.7)	2 (1.1)	.68^f^
Participants experiencing pressure for social security leave, n (%)		2 (1.1)	4 (2.2)	.69^f^
Participants facing financial reduction with significant impact on the monthly family budget, n (%)		3 (1.7)	5 (2.8)	.72^f^
**Participants’ educational level (n=356),** **n (%)**				.009^c^
	Elementary school	12 (6.8)	5 (2.8)	—
	High school	85 (48.3)	65 (36.1)	—
	University	47 (26.7)	57 (31.7)	—
	Postgraduate education	32 (18.2)	53 (29.4)	—

^a^Interquartile range: 2 (1-3).

^b^Nonparametric Mann–Whitney test.

^c^Chi-square test.

^d^Not applicable.

^e^Interquartile range: 2 (1-2).

^f^Fisher exact test.

Most of the patients who answered the questionnaire were in remission from GTD (210/356 after spontaneous remission and 67/356 after chemotherapy; Table 3). RCP felt safer and more confident at the time of diagnosis of GTD (84/176) than NRCP (53/180; *P*=.001). Concordantly, NRCP reported more frequently (55/180) than RCP (32/176; *P*=.007) that what most negatively affected them at the time of diagnosis was the fact that doctors did not explain GTD. RCP more frequently received all treatment from the Brazilian public health system than NRCP (106/176 vs 60/180; *P*<.001). Also, there was more pronounced migration during therapy from the private health system to the Brazilian public health system among RCP (29/176) than among NRCP (16/180; *P*<.001). Although RCP and NRCP both reported similar sexual activity (*P*=.37), notably less than half of the NRCP (86/180) used a hormonal contraceptive method compared with the majority of RCP (121/176; *P*<.001).

Table 4 presents the netnography characteristics of participating members of FBGTD. Of all, 89% (317/356) patients accessed the internet preferentially from home using mobile phones. Facebook and WhatsApp were the social networks most frequently used by patients (327/356, 92%). Instagram use was more frequent among NRCP (114/180) than among RCP (92/176, *P*=.03). Facebook groups of interest among these patients were those that provided answers to questions about the disease and treatment options (246/356, 69%) and that provided exchange of experiences among people who have a problem or a similar interest (274/356, 77%).

NRCP found FBGTD through Web searches (139/180 vs 85/176, *P*<.001; Table 5) while RCP were more likely to be referred to FBGTD through health assistants (64/176 vs 32/180, *P*<.001; Table 5). NRCP were more likely to be on FBGTD longer than 6 months (118/180 vs 95/176, *P=*.03; Table 5) and to raise more questions about GTD treatment (107/180) than RCP (84/176, *P*=.01; Table 5).

**Table 3:** Clinical and psychosexual characteristics of women with gestational trophoblastic disease and members of Facebook group of the Brazilian Association of gestational trophoblastic disease.

Variables		Treated at reference centers (n=176)	Not treated at reference centers (n=180)	*P* value
**At what point in the GTD^a^ treatment do you find yourself?, n (%)**				.22^b^
	Waiting for molar evacuation	2 (1.1)	1 (0.6)	—^c^
	Postmolar follow-up	30 (17.0)	33 (18.3)	—
	Chemotherapy	9 (5.1)	4 (2.2)	—
	Spontaneous remission	96 (54.5)	114 (63.3)	—
	Remission after chemotherapy	39 (22.2)	28 (15.6)	—
**Regarding this^d^ statement, how do you evaluate your experience when you received the diagnosis of GTD?, n (%)**				.001^b^
	Strongly agree	84 (47.7)	53 (29.4)	—
	Agree	40 (22.7)	49 (27.2)	—
	Neither agree or disagree	0 (0.0)	4 (2.2)	—
	Disagree	14 (8.0)	15 (8.3)	—
	Strongly disagree	38 (21.6)	59 (32.8)	—
**What contributed to the negative experience when you were diagnosed with GTD?, n (%)**				
	The doctors used complicated words, which made it difficult for me to understand	12 (6.8)	20 (11.1)	.16^e^
	The doctors have not explained my disease at all	32 (18.2)	55 (30.6)	.007^e^
	Although the doctors explained to me about GTD, I did not understand	10 (5.7)	19 (10.6)	.09^e^
	The doctors did not explain how my treatment should be	35 (19.9)	43 (23.9)	.36^e^
	The doctors did not explain where my treatment should be	30 (17.0)	35 (19.4)	.56^e^
	I did not get to schedule my appointment quickly in the reference center	7 (4.0)	9 (5.0)	.64^e^
**During postmolar follow-up, how are you avoiding pregnancy?^f^ n (%)**				
	Hormonal methods	121 (68.8)	86 (47.8)	<.001^b^
	Condom	38 (21.6)	53 (29.4)	.09^b^
	Behavioral contraceptive methods	19 (10.8)	29 (16.1)	.14^e^
**Regarding the location of my treatment, n (%)**				<.001^b^
	I started in the public health system, where I underwent all my treatment	106 (60.2)	60 (33.3)	—
	I started in the public health system, but then I went to the private health system where I underwent all my treatment	1 (0.6)	7 (3.9)	—
	I started in the private health system, but then I went to the public health system where I underwent all my treatment	29 (16.5)	16 (8.9)	—
	I started in the private health system, where I underwent all my treatment	10 (5.7)	76 (42.2)	—
	I am undergoing my treatment at both services (public and private)	30 (17.0)	21 (11.7)	—
**How is the relationship with your partner after the diagnosis of GTD? (N=355)^g^, n (%)**				.48^b^
	GTD made us nearer	62 (35.4)	47 (26.1)	—
	Our relationship is the same	87 (49.7)	105 (58.3)	—
	I feel that my relationship is getting weaker	15 (8.6)	14 (7.8)	—
	I broke up my relationship by my own initiative	6 (3.4)	7 (3.9)	—
	My partner left me	3 (1.7)	3 (1.7)	—
	I had no stable relationship	2 (1.1)	4 (2.2)	—
**How has your sex life been since you were diagnosed with GTD?, n (%)**				.37^e^
	Similar	112 (63.6)	125 (69.4)	—
	Better	12 (6.8)	6 (3.3)	—
	Worse	38 (21.6)	33 (18.3)	—
	I do not have a sex life	14 (8.0)	16 (8.9)	—
	I have at least one child	82 (46.6)	93 (51.7)	.34^e^
**Would get pregnant again?, n (%)**				.37^e^
	Yes	113 (64.2)	127 (70.6)	—
	Maybe	32 (18.2)	24 (13.3)	—

^a^GTD: gestational trophoblastic disease.

^b^Likelihood ratio test.

^c^Not applicable.

^d^ *When I first received the diagnosis of molar pregnancy, known as hydatidiform mole or gestational trophoblastic disease (GTD), my attending team informed me in a accessible (understandable) and enlightening way what was all this disease about, where and what would be my treatment (and follow-up). I started my treatment quickly after that and felt myself safe and confident.*

^e^Chi-square test.

^f^More than one option could be opted.

^g^One patient did not respond to this question.

**Table 4:** Netnography characteristics of patients with gestational trophoblastic disease and members of Facebook group of the Brazilian Association of gestational trophoblastic disease who participated in this study.

Variables		Treated at reference centers (n=176)	Not treated at reference centers (n=180)	*P* value
**Which location do you access internet preferentially from?^a^, n (%)**				
	Home	171 (97.2)	174 (96.7)	.79^b^
	Work	42 (23.9)	54 (30)	.19^b^
**Most of the time, you access the internet through which mode?, n (%)**				
	Desktop	9 (5.1)	10 (5.6)	.85^b^
	Mobile phone or Smartphone	158 (89.8)	159 (88.3)	.66^b^
	Notebook or Laptop	8 (4.5)	10 (5.6)	.66^b^
**Which social networks do you participate besides Facebook?^a^, n (%)**				
	Blog	5 (2.8)	3 (1.7)	.50^c^
	Twitter	10 (5.7)	17 (9.4)	.18^b^
	Instagram	92 (52.3)	114 (63.3)	.03^b^
	WhatsApp	162 (92)	165 (91.7)	.90^b^
	G+	11 (6.3)	13 (7.2)	.71^b^
	Snapchat	14 (8.0)	25 (13.9)	.07^b^
**What kind of groups do you seek in Facebook?^a^, n (%)**				
	That provide me emotional support	38 (21.6)	37 (20.6)	.81^b^
	That answer my questions about the disease and treatment options	117 (66.5)	129 (71.7)	.29^b^
	That promote leisure activities	47 (26.7)	46 (25.6)	.81^b^
	That recommend specialized services and professionals	61 (34.7)	49 (27.2)	.13^b^
	That provide exchange of experiences among people who have a problem or similar interest	133 (75.6)	141 (78.3)	.54^b^

^a^More than one answer was possible.

^b^Chi-square test.

^c^Fisher exact test.

**Table 5:** “Tropho-netnography” evaluation of the women participating in the study.

Variables			Treated at reference centers (n=176)	Not treated at reference centers (n=180)	*P* value^a^
**How did you find the Facebook group of the Brazilian Association of Gestational Trophoblastic Disease?, n (%)**					
		Search sites and pages	85 (48.3)	139 (77.2)	<.001
		Friend referral	11 (6.3)	9 (5.0)	.61
		Patient referral	19 (10.8)	6 (3.3)	.006
		Health assistant referral	64 (36.4)	32 (17.8)	<.001
**How often do you access this group on Facebook? (n=355), n (%)**					.02
		Daily	122 (69.7)	103 (57.5)	—^b^
		Weekly	36 (20.6)	61 (34.1)	—
		Rarely	17 (9.7)	15 (8.4)	—
**How long have you been in this group on Facebook?, n (%)**					.03
		≤6 months	81 (46)	62 (34.4)	—
		> 6 months	95 (54)	118 (65.6)	—
**What are the main reasons that lead to follow this group** **on Facebook?^c^, n (%)**					
		I find emotional and psychological support in the group	87 (49.4)	90 (50.0)	.91
		I get more information about the disease and treatment	138 (78.4)	148 (82.2)	.36
		I receive directions for specialized professional and reference center for treatment and follow-up	68 (38.6)	78 (43.3)	.37
		I enlarge my network of online friends	4 (2.3)	7 (3.9)	.38
		I have the opportunity to interact with patients with gestational trophoblastic disease	103 (58.5)	102 (56.7)	.72
**Have you published or liked something in this group on Facebook?, n (%)**					
	**If NO, why not?^c^**		16 (9.1)	18 (10.0)	.77
		I do not feel comfortable	4 (25)	3 (16.7)	.68
		I would rather just watch	10 (62.5)	11 (61.1)	.93
		I think it is a lot of exposure	1 (6.3)	3 (16.7)	.60
	**If YES, what were the post subjects?^c^**		160 (90.9)	162 (90.0)	
		Ask questions about my treatment	84 (52.5)	107 (66.0)	.01
		Give support to someone who feels discouraged	78 (48.8)	75 (46.3)	.66
		Share the success of my treatment	73 (45.6)	76 (46.9)	.82
		Receive comfort and words of encouragement	35 (21.9)	41 (25.3)	.47
		I liked a publication I found appropriate	116 (72.5)	103 (63.6)	.09
**How do you feel about the advice posted by doctors in this group on Facebook? (n=354), n (%)**					.62^d^
		Completely safe	146 (83.9)	156 (86.7)	—
		Partially safe	26 (14.9)	21 (11.7)	—
		I have no opinion	2 (1.1)	3 (1.7)	—
		Partly unsafe^e^	1 (0.0)	0 (0.0)	—
		Totally unsafe^e^	1 (0.0)	0 (0.0)	—
**How do you feel about the advice posted by the patients in this group in Facebook?, n (%)**					.81^d^
		Completely safe	57 (32.4)	57 (31.7)	—
		Partially safe	93 (52.8)	99 (55)	—
		No opinion	14 (8.0)	16 (8.9)	—
		Partly unsafe	9 (5.1)	7 (3.9)	—
		Totally unsafe	3 (1.7)	1 (0.6)	—
**In addition to specialized physicians, what other professional would you like to actively participate in this group on Facebook?, n (%)**					
		Social worker (N=356)	30 (17.0)	39 (21.7)	.27
		Lawyer (N=356)	11 (6.3)	10 (5.6)	.78
		Nurse (N=356)	31 (17.6)	27 (15.0)	.50
		Psychologist (N=355)	126 (72.0)	131 (72.8)	.87
		I do not see the need for other professionals (N=356)	37 (21.0)	34 (18.9)	.61

^a^Chi-square test.

^b^Not applicable.

^c^More than one answer was possible.

^d^Likelihood ratio test.

^e^These numbers were excluded for comparison because of the small frequency.

## Discussion

This netnographic study of GTD patients in the FBGTD indicates that GTD patients find a moderated online community to be an important and trustworthy source of peer support and medical advice. Patients drew psychoaffective support from the online interactions and an improved understanding of the disease and its management. These benefits were most evident among NRCP.

GTD is a rare disease, and outcomes of GTD treatment have improved worldwide with the establishment of reference centers, with exceptional rates of cure and of the ability to preserve fertility [19]. Further distance between the residence of the patients with GTD and the referral center worsens the prognosis of this disease, likely related to delay in the diagnosis, and is associated with more advanced stages of GTN at diagnosis and higher rates of incomplete follow-up [20]. The internet can mitigate these effects, acting as a medium for the diffusion of knowledge about malignant disease [21], including GTD, not only among physicians but also among patients, helping them find specialized services, promoting a rich exchange of information, and, in selected cases, promoting personalized guidance through electronic evaluations using private emails [22].

In Brazil there are 236 million mobile phones in use (113.8/100 inhabitants) [23], which could explain mobile phones as the most common way to access the FBGTD. With the increase in mobile phone ownership, health care providers have become interested in integrating the use of mobile phones in the care of chronic conditions such as HIV, diabetes, hypertension, and asthma [24-28]. Mobile phones are portable, capable of receiving and transmitting data, and “always on.” They also offer health care providers the unique ability to connect with hard-to-reach populations that might otherwise not have access to health care services [29]. Our data showing that GTD patients most commonly have accessed the FBGTD via mobile phone may represent an opportunity to develop apps, monitored by specialized physicians, to facilitate and improve hCG follow-up. Such an innovation would enable the GTD patients to be more active agents in managing their own health care.

Facebook is the most widely used social media platform and has been recognized as a tool to support patients [30,31] and pregnant women [32]. Some medical societies have issued recommendations on how to appropriately use Facebook and other social media. These recommendations, aimed at physicians and patients, highlight how to preserve confidentiality and to avoid medicolegal complications [33]. The Brazilian FBGTD page was created 5 years ago by Drs MV and AB, directors of Brazilian Association of GTD, observing these recommendations. Since then, they and other Brazilian Association of GTD physicians have posted advice for individual patients (answering individual specific questions) as well as for groups of patients. It is important to emphasize that they encourage patients to have a face-to-face appointment at the closest BRC, advising that the online recommendations should not replace a thorough personal consult (medical history, physical exam, images, and laboratory evaluation). However, some patients reside in regions distant from a BRC, and FBGTD is the only means through which such patients can access an expert in GTD.

Of all, 98% (349/354) patients declared they felt safe reading the posts from the FBGTD physicians. Web-based interactions used simple, understandable, warm, and empathetic language and facilitated an effective doctor-patient relationship. This could be one of the reasons that a quarter of the patients answered the survey only after Dr AB initiated an invitation to participate.

Based on the success of the FBGTD, similar efforts have been launched for other diseases. For example, in Porto Alegre in Brazil, a closed Facebook group similar to FBGTD was created in 2018 as an education platform to involve adolescent renal transplant patients [34]. This new group facilitates the expression of feelings and contributes to increased self-esteem, reduced anxiety, and increased adherence to the treatment plan.

It should be noted that women with GTD and higher educational level are more likely to have follow-up outside a BRC. In general, these women have greater resources and do not undergo treatment under the Brazilian public health system. However, we observed that 64% (29/45) of these women, who initially sought private health assistance, migrated to the Brazilian public health system to be seen in a BRC, showing the quality of these specialized services [10,12]. In addition, patients treated outside a BRC are more likely to express doubts about GTD and less able to interpret the information provided by their doctors. This may be related to fundamental gaps in GTD understanding on the part of gynecologists and oncologists not associated with a referral center [22]. Moreover, at a BRC, care is provided exclusively to patients with GTD and, therefore, the physicians can spend more time discussing the disease and its proper follow-up. This is illustrated in the significant difference in the adoption of effective hormonal contraception in the postmolar follow-up, fundamental to providing reliable hormonal surveillance [35,36] and much more evident among the women followed at a BRC.

A striking netnography difference in this study showed that patients with GTD treated outside a BRC accessed the FBGTD for a longer duration than those who were treated a BRC. This is most likely because these women had less understanding of the disease and had not had the opportunity to get their concerns addressed by a doctor specializing in GTD.

One of the limitations of this study is the nature of nonprobabilistic sampling, which can compromise the quantitative data analysis. Consequently, our results may not be easily extrapolated to other GTD populations. Another issue that should be emphasized is that all responses were provided by the patients, and there is no way to verify the accuracy of the information provided. In addition, there may be differences between women who use social media or choose to participate in social media questionnaires and those who do not. The issues of patient response and patient self-selection could produce both recall bias and selection bias. On the other hand, we gained important insights about the Web-based doctor-patient relationship among patients with GTD. The use of an understandable and warm language, even without a face-to-face meeting between a doctor and a patient, can establish a safe and confident doctor-patient relationship. This Web-based relationship may be important in strengthening the strategies related to the education of GTD patients on a large scale, which may be particularly important in countries with a large territorial area, leading to difficulties in obtaining quality health care.

In this new Web-based social media era, physicians and other health professionals should understand that patients asking questions in an internet environment are trying to make sense of their treatment. A Facebook page headed by experienced and knowledgeable professionals, as the Brazilian FBGTD, can play an important role to correct misinformation [15,32] and be a valuable tool for medical learning [37] and supporting patients.

## Appendix

Multimedia Appendix 1 Questionnaire.

## Abbreviations

BRC: Brazilian gestational trophoblastic disease reference center
FBGTD: Facebook group of the Brazilian Association of gestational trophoblastic disease
GTD: gestational trophoblastic disease
GTN: gestational trophoblastic neoplasia
NRCP: nonreference center patients
RCP: reference center patients
SMS: short message service
