# Supplementary material for: Experience With the Use of an Online Community on Facebook for Brazilian Patients With Gestational Trophoblastic Disease: Netnography Study
Source: J Med Internet Res. 2018 Sep 24;20(9):e10897. doi: 10.2196/10897 (PMC6231805; doi:10.2196/10897)
Supplement: Multimedia Appendix 1 [file jmir_v20i9e10897_app1.pdf]

Invitation

We are inviting you, member of the ABDTG Facebook® group (Associação Brasileira de Doença Trofoblástica Gestacional), to be part of a UNIFESP (Universidade Federal de São Paulo) scientific research where we try to have a better understanding of the profile and way of thinking of the attendees of this group. Your participation must be **VOLUNTARY** and will **NOT BE PAID**.

It consists of a questionnaire with 33 online questions that takes approximately 10 minutes to answer. The data collected will be confidential and later analyzed with the objective of knowing the functionality of this social group and ways to improve it. Again, **WE GUARANTEE** the confidentiality of the information you trust us with.

Online Questionnaire

UNDERSTANDING THE ONLINE CULTURAL ASPECTS OF THE GESTATIONAL TROPHOBLASTIC DISEASE (GTD) PATIENTS.

ONLINE CULTURAL PROFILE OF THE GESTATIONAL TROPHOBLASTIC DISEASE (GTD) PATIENTS.

Internet relationship *and habits.*

1. From which location do you access the internet? You may choose more than an option.

- ❑ Home
- ❑ Work
- ❑ School
- ❑ Lan house *or internet café*
- ❑ Others (please specify)

2. How do you access the internet most of the time?

- ❑ Cellphone / *Smartphone*
- ❑ Desktop
- ❑ Notebook / *Laptop*
- ❑ Tablet
- ❑ *Others (please specify)*

3. What other online social networks do you participate, besides Facebook? You may choose more than an option.

- ❑ Blog
- ❑ Twitter
- ❑ Instagram
- ❑ WhatsApp
- ❑ Google+ (G+)
- ❑ Snapchat
- ❑ *Youtube channel*
- ❑ Others (please specify)

4. What kind of groups do you seek in Facebook? *You may choose more than an option.*

- ❑ That provide me emotional support.
- ❑ That answer my questions about the disease and treatment options.
- ❑ That lead me to recreation.
- ❑ That recommends specialized professionals and services.
- ❑ That provide an experience exchange between other patients with the same disease or interests.
- ❑ Others (please specify).

Disease record

5. **"When I first received the diagnosis of Molar Pregnancy, known as Hydatiform Mole or Gestational Trophoblastic Disease (GTD), my attending team informed me in a accessible (*understandable*) and enlightening way what was all this disease about, where and what would be my treatment (*and follow-up*). I started my treatment quickly after that and felt myself safe and confident."**

Comparing what is stated above with your **own experience**, choose one of the following options:

- ❑ Strongly disagree (proceed to question 5.1)
- ❑ Disagree (proceed to question 5.1)
- ❑ Neither agree or disagree(follow to question 6)
- ❑ Agree (proceed to question 5.1)
- ❑ Strongly agree (proceed to question 6)

5.1 . From the following options, which one better explain your last answer? You may choose more than an option.

- ❑ My medical team used complicated words and expressions that compromised my understanding.
- ❑ My medical team have not explained my disease at all.

- ❑ My medical team explained my situation, but I did not understand.
- ❑ They did not explained **how** my treatment should be.
- ❑ They did not explained **where** my treatment should be.
- ❑ They did not sent me for follow-up.
- ❑ My treatment has not started quickly enough.
- ❑ Others possible explanation, please specify.

6. How is your β-hCG now?

- ❑ Negative (less or equal 5). Follow to question 6.1
- ❑ Positive (more than 5). Follow to question 6.2
- ❑ I do not know or have not started yet. Follow to question 7

6.1 Choose one of the following options:

- ❑ I am in my **first negative result** after the mole disease removal .
- ❑ I have already **3 or more negative results** after the mole disease removal.
- ❑ I am in my **first negative result** after chemotherapy.
- ❑ I have already **3 or more results** after chemotherapy.
- ❑ Others, please specify.

6.2 Choose one of the following options:

- ❑ It is positive and rising - I have NOT removed the mole disease.
- ❑ It is positive and rising - It is my **first positive result** since the mole disease removal.
- ❑ It is positive and rising - It is my **first positive result** since the beginning of chemotherapy.
- ❑ It is positive, but decaying - after I removed the disease.
- ❑ It is positive, but decaying - after I begun chemotherapy.
- ❑ Others, please specify.

7. Does someone in your family had MOLAR PREGNANCY?

- ❑ No, nobody.
- ❑ Mother
- ❑ Maternal grandmother
- ❑ Paternal grandmother
- ❑ Sister
- ❑ Maternal aunt
- ❑ Paternal aunt
- ❑ Maternal first degree cousin
- ❑ Paternal first degree cousin

8. How have you met the Facebook group of Associação Brasileira de Doença Trofoblástica Gestacional (Gestational Trophoblastic Disease Brazilian Association)?

- ❑ Search sites and pages
- ❑ Friend referral
- ❑ Patient referral
- ❑ Health assistant referral
- ❑ Others, please specify.

9. How often do you access the group posts?

- ❑ Daily
- ❑ Weekly
- ❑ Rarely

10. How long have been a member of this group?

- ❑ More than 6 months
- ❑ Less than 6 months

(item parecido com questão 4)

11. What are the main reasons that lead to follow this group? You may choose more than an option.

- ❑ I find emotional and psychological support.
- ❑ I obtain more information about the disease and treatment options.
- ❑ I receive directions for specialized professionals and referral centers for treatment and follow-up.
- ❑ I enlarge my virtual friend circle.
- ❑ I have the opportunity to interact with other patients with the same medical condition.
- ❑ Others, please specify.

12. Have you published or “liked” any post in the group?

- ❑ No. (proceed to question 12.1)
- ❑ Yes. (proceed to question 12.2)

12.1 What is the reason (or reasons) behind your decision to not post anything? You may choose more than an option.

- ❑ I do not feel comfortable.
- ❑ I prefer to just observe.
- ❑ In my opinion it is too much openness.
- ❑ I am afraid of being judged.
- ❑ I believe is illegal to post my results on Facebook.
- ❑ Others, please specify.

12.2 What kind of post have you made? You may choose more than an option.

- ❑ I have my doubts about the treatment.
- ❑ I wanted to cheer up/give support to someone who was feeling discouraged.
- ❑ I wanted to share the success of my treatment.
- ❑ I wanted to receive comfort and words of encouragement.
- ❑ I commented positively a post with which agreed.
- ❑ I commented negatively a post with which I did not agreed.
- ❑ Others, please specify.

13. How do you feel about the orientations posted by DOCTORS?
    - ❑ Completely unsafe
    - ❑ Partially unsafe
    - ❑ I have no opinion
    - ❑ Partially safe
    - ❑ Completely safe

14. How do you feel about the orientations posted by OTHER MEMBERS of the group?
    - ❑ Completely unsafe
    - ❑ Partially unsafe
    - ❑ I have no opinion
    - ❑ Partially safe
    - ❑ Completely safe

15. Besides specialized physicians, which other professionals would like to see actively participating in the group? You may choose more than an option.
    - ❑ Social Assistant
    - ❑ Lawyer / Attorney
    - ❑ Nurse
    - ❑ Nutritionist
    - ❑ Psychologist
    - ❑ I do not feel the need of any other professionals.
    - ❑ Other, please specify.

16. Would you rather be in a closed group only with the physicians and patients from your place of treatment?
    - ❑ No
    - ❑ Yes
    - ❑ Maybe

17. How is your sexual life since the diagnosis of molar disease?
    - ❑ Same as before.
    - ❑ It improved
    - ❑ It got worse
    - ❑ I do not have a sexual life

18. How is the relationship with your partner after the diagnosis of molar disease?

- ❑ I broke up my relationship by my own initiative.
- ❑ My partner left me.
- ❑ I had no stable relationship.
- ❑ The disease made us nearer.
- ❑ I keep my relationship as before.
- ❑ I feel that my relationship is getting weaker.

19. How are you preventing pregnancy?

- ❑ Birth control pill
- ❑ Birth control shot
- ❑ Withdrawal (pull out method)
- ❑ Fertility awareness method
- ❑ Emergency contraceptive pill
- ❑ Condom
- ❑ Others, please specify

20. Do you have children?

- ❑ No
- ❑ Yes

21. Would get pregnant again?

- ❑ No
- ❑ Yes
- ❑ Maybe

22. About your treatment (you may choose more than an option):

- ❑ It begun in the public healthcare system (SUS - Sistema Único de Saúde) and I am still following in it.
- ❑ It begun in the public healthcare system, but I changed to private care.
- ❑ It begun in the private care, but I changed to the public healthcare system.
- ❑ I do my follow-ups in both health systems.

23. Currently, do you follow up in one of the Reference Centers for GTD Treatment?

- ❑ No (proceed to question 24)
- ❑ Yes (proceed to question 23.1)

23.1 Have you begun your follow-up in one of the Reference Centers by referral of one the members from the Facebook group?

- ❑ No (proceed to question 23.3)
- ❑ Yes (proceed to question 23.2)

23.2 How long have you waited for your first appointment in the Reference Center after the referral of one of the members of the facebook group:

- ❑ Up to 1 day
- ❑ From 2 to 3 days
- ❑ From 4 to 7 days
- ❑ From 8 to 10 days
- ❑ From 11 to 14 days
- ❑ Others (proceed to question 23.4)

23.3 How long have you waited for the beginning of your treatment in the reference Center:

Up to 1 day

- ❑ From 2 to 3 days
- ❑ From 4 to 7 days
- ❑ From 8 to 10 days
- ❑ From 11 to 14 days
- ❑ Others (proceed to question 23.4)

23.4 How do you feel about the medical care offered you by the Reference Center?

- ❑ Completely unsatisfied.
- ❑ Partially unsatisfied
- ❑ I do not have an opinion
- ❑ Partially satisfied
- ❑ Completely satisfied

Your profile

24. How old are you?

25. How tall are you?

26. What was your weight when you first discovered the disease?

27. What is your current weight?

28. What is your color/race?

- ❑ White/Caucasian
- ❑ Black/Afro
- ❑ Yellow/Asian
- ❑ Native
- ❑ I do not want to declare my race
- ❑ Other, please specify

29. What is your educational degree?

- ❑ Primary Education (elementary school)
- ❑ Secondary Education (high school)
- ❑ College
- ❑ Post-graduation

30. How many people live at your place, besides you?

(number number)

31. How many people contribute for the financial income of your home?

(number number)

32. What is the CITY, STATE and COUNTRY you are currently living in?

33. Do you have any financial income activity?

- ❑ No
- ❑ Yes (proceed to question 33.1)

33.1 Do you have or had any labor difficulties to perform your treatment?

- ❑ No (proceed the ending A)
- ❑ Yes (proceed to question 33.2)

33.2 Concerning your labor and your treatment, which of the following difficulties are you facing or had faced? You may choose more than an option.

- ❑ My medical certificates had been refused.
- ❑ Inappropriate salary or leave discounts
- ❑ Pressure for Social Security leave.
- ❑ Physical or emotional discomfort to perform usual activities.
- ❑ Resignation
- ❑ Financial reduction with significant impact in the monthly family budget.
- ❑ Others, please specify.

Ending A:

We appreciate immensely your cooperation, your tolerance and patience. Be certain that this little minutes you have just offered us were extremely useful for the improvement of something splendid that we define as a “mission of doing good” and, without your help, it would be impossible!

Thank you!

Ending B: (in case patient that patient has not agreed to participate)

Despite the situation, bravery and conviction are decisive factors for reaching success. We appreciate immensely your cooperation!

Thank you!
